# An efficient system composed of maize protoplast transfection and HPLC–MS for studying the biosynthesis and regulation of maize benzoxazinoids

**DOI:** 10.1186/s13007-019-0529-2

**Published:** 2019-11-28

**Authors:** Lei Gao, Guojing Shen, Lingdan Zhang, Jinfeng Qi, Cuiping Zhang, Canrong Ma, Jing Li, Lei Wang, Saif Ul Malook, Jianqiang Wu

**Affiliations:** 1 Department of Economic Plants and Biotechnology, Yunnan Key Laboratory for Wild Plant Resources, Kunming Institute of Botany, Chinese Academy of Sciences, Kunming, 650201 China; 2 School of Biological Science, Yunnan University, Kunming, 650091 China

**Keywords:** Maize, Benzoxazinoids, Protoplasts, Transient gene expression, Transcription factor, HPLC–MS

## Abstract

**Background:**

Insect herbivory poses a major threat to maize. Benzoxazinoids are important anti-insect secondary metabolites in maize, whose biosynthetic pathway has been extensively studied. However, yet little is known about how benzoxazinoids are regulated in maize, partly due to lack of mutant resources and recalcitrance to genetic transformation. Transient systems based on mesophyll- or cultured cell-derived protoplasts have been exploited in several plant species and have become a powerful tool for rapid or high-throughput assays of gene functions. Nevertheless, these systems have not been exploited to study the regulation of secondary metabolites.

**Results:**

A protocol for isolation of protoplasts from etiolated maize seedlings and efficient transfection was optimized. Furthermore, a 10-min-run-time and highly sensitive HPLC–MS method was established to rapidly detect and quantify maize benzoxazinoids. Coupling maize protoplast transfection and HPLC–MS, we screened a few genes potentially regulating benzoxazinoid biosynthesis using overexpression or silencing by artificial microRNA technology.

**Conclusions:**

Combining the power of maize protoplast transfection and HPLC–MS analysis, this method allows rapid screening for the regulatory and biosynthetic genes of maize benzoxazinoids in protoplasts, before the candidates are selected for in planta functional analyses. This method can also be applied to study the biosynthesis and regulation of other secondary metabolites in maize and secondary metabolites in other plant species, including those not amenable to transformation.

## Background

Maize (*Zea mays*) is one of the world’s most widely cultivated crops, providing food to a large portion of the world population, and is used as animal feed as well a source of biofuel. The rapid development of high-throughput sequencing has greatly boosted the field of maize genomics and genetics [1–3]. Although an increasing number of genes have been characterized, the functions of the majority of genes in maize genome remain unclear. In vitro biochemical assays can be done to study the functions of genes encoding enzymes. Nevertheless, knock-in and knock-out/-down lines are highly desired for examining gene functions *in planta,* including maize. To date, maize still lacks mutant resources and it is relatively recalcitrant to transformation, hindering functional genomics of maize.

**Fig. 1:**
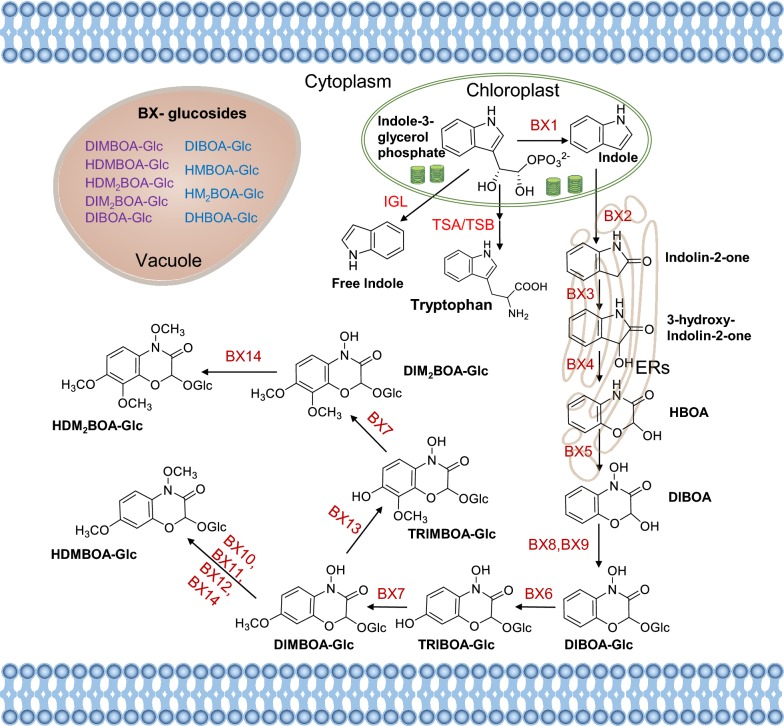
Biosynthesis and compartmentalization of benzoxazinoids in maize. The biosynthesis of benzoxazinoids starts from the conversion of the indole-3-glycerol phosphate to indole in the chloroplasts, which is catalyzed by BX1. The four cytochrome P450-dependent monooxygenases, BX2 to BX5, function consecutively to synthesize DIBOA. UDP-glucosyltransferase BX8 or BX9 uses DIBOA to form DIBOA-Glc in the cytoplasm. DIBOA-Glc is further hydroxylated by the BX6 and subsequent methylated by the BX7 to yield DIMBOA-Glc. The DIMBOA-Glc is then converted to HDMBOA-Glc by the O-methyltransferases, BX10, BX11, BX12, or BX14. BX13 catalyzes the conversion of DIMBOA-Glc to TRIMBOA-Glc, and the latter can be O-methylated by BX7 to form DIM_2_BOA-Glc, which can be further methylated by BX14 to generate HDM_2_BOA-Glc. Benzoxazinoids are mainly stored as glucosides in the vacuoles, and upon injury, the glucosides are enzymatically hydrolyzed into the respective aglucones and sugars

In nature, almost all plants suffer from insect attack. To survive, plants have evolved a diverse repertoire of secondary metabolites to defend themselves against insects [4]. In maize, benzoxazinoids are a class of indole-derived secondary metabolites, which are the major defense chemicals against insect herbivores [5–7]. Moreover, benzoxazinoid biosynthesis mutants also exhibit increased susceptibility to fungal infection, indicating the role of benzoxazinoids in maize immunity to diseases [8, 9]. Decades of study has resulted in identification of a nearly complete biosynthetic pathway of benzoxazinoids in maize [10–14]. The biosynthesis of benzoxazinoids starts in the plastids, in which the conversion of the shikimate pathway-derived indole-3-glycerol phosphate is converted to indole, and this reaction is catalyzed by the indole-3-glycerol phosphate lyase (benzoxazinoneless 1, BX1) (Fig. 1) [14]. The four cytochrome P450 monooxygenases, BX2 to BX5, function consecutively to synthesize DIBOA (2,4-dihydroxy-1,4-benzoxazin-3-one) by oxidizing indole and sequentially incorporating oxygen atoms. The unstable bioactive DIBOA aglucone is stabilized by glucosylation catalyzed by the UDP-glucosyltransferase BX8 and BX9 to form DIBOA-Glc in the cytoplasm (Fig. 1) [15]. DIBOA-Glc is further hydroxylated by the 2-oxoglutarate-dependent dioxygenase BX6 and subsequent methylated by the *O*-methyltransferase BX7 to yield DIMBOA-Glc (2,4-dihydroxy-7-methoxy-1,4-benzoxazin-3-one glucoside) in the cytosol [16], and DIMBOA-Glc is then converted to HDMBOA-Glc (2-hydroxy-4,7-dimethoxy-1,4-benzoxazin-3-one glucoside) by *O*-methylation, which is catalyzed by a group of three homologous *O*-methyltransferases, BX10, BX11, BX12, which were formerly designated BX10a, BX10b, and BX10c, respectively, or BX14 [10, 12]. Recently, the formation of two maize 8-*O*-methylated benzoxazinoids DIM_2_BOA-Glc (2,4-dihydroxy-7,8-dimethoxy-1,4-benzoxazin-3-one glucoside) and HDM_2_BOA-Glc (2-dihydroxy-4,7,8-trimethoxy-1,4-benzoxazin-3-one glucoside) has been elucidated: BX13, a 2-oxoglutarate-dependent dioxygenase, catalyzes the conversion of DIMBOA-Glc to TRIMBOA-Glc (2,4,7-trihydroxy-8-methoxy-1,4-benzoxazin-3-one glucoside), and the latter can be *O*-methylated by BX7 to form DIM_2_BOA-Glc, which can be further methylated by another *O*-methyltransferase BX14 to generate HDM_2_BOA-Glc (Fig. 1) [10].

Even though almost all the benzoxazinoid biosynthetic genes have been identified, very little is known about how benzoxazinoid biosynthesis is regulated. Large variations of benzoxazinoid contents have been detected among different maize lines [12]. Using a mapping population derived from the inbreed lines B73 and Mo17, whose benzoxazinoid levels are low and high, respectively, Zheng et al. [17] found that that a 3.9-kb *cis*-element located approximately 140 kb upstream of *BX1* gene enhances *BX1* transcript levels in Mo17, leading to its highly benzoxazinoid content. Moreover, exogenously supplementation of jasmonic acid (JA) to maize elevated benzoxazinoid contents [18], indicating that JA signaling is part of the regulatory network of benzoxazinoid biosynthesis. Using the maize line A188, Qi et al. [19] showed that lepidopteran insect *Mythimna separata* feeding on maize activated transient bursts of several phytohormones JA, salicylic acid, abscisic acid, and ethylene, as well as increased benzoxazinoid levels, suggesting the possible involvement of these phytohormones in regulating benzoxazinoids. Transcriptomic analyses and association studies have also revealed many potential benzoxazinoid regulators [19–21]. Given the shortage of mutants and efficient transformation systems, a relatively high-throughput method is needed for investigating the functions of candidate benzoxazinoid regulators and metabolism genes in maize.

Plant protoplast isolation was first achieved more than half a century ago using the root tips of tomato (*Solanum lycopersicum*) seedlings [22]. Leaves [23], fruits [24] and stems [25] have been used as the sources for isolating protoplasts. Macromolecules, such as DNA, can be delivered into protoplasts using polyethylene glycol (PEG)-mediated [26] or electroporation-mediated transfection [27], and microinjection has also been applied in protoplasts [28]. In recent years, mesophyll protoplast-based transient expression assays have been developed in Arabidopsis (*Arabidopsis thaliana*) [23], tobacco (*Nicotiana tabacum*) [29], rice (*Oryza sativa*) [30], and maize (*Zea mays*) [31], as well as in an increasing number of other species, such as *Magnolia* [32] and *Liriodendron* [33]. These protoplast transfection systems have become indispensable tools for studying gene functions in a high-throughput and cost-effective manner.

Although protoplast transfection systems are commonly used for studying protein subcellular localization [34], promoter activity [35], protein–protein interactions [36], and signal transduction [37], these systems have rarely been exploited for identifying gene function in secondary metabolite biosynthesis or regulation. Taking advantages of the high throughput of maize protoplast transfection system and the high specificity and sensitivity of HPLC–MS (high performance liquid chromatography–mass spectrometry) analysis, we developed a platform that can be used for studying the genes putatively function in benzoxazinoid biosynthesis or regulation, and the whole procedure from protoplast isolation to obtaining results takes only 2 days. Similar methods can be developed to investigate the biosynthetic genes and regulators of specific secondary metabolites in other plant species, including those whose transformation systems are unavailable or difficult.

## Materials and methods

### Plant materials

The seeds of maize (*Zea mays*) inbred lines B73 and W22 and the *bx2::Ds* mutant (gene identifier GRMZM2G085661; Ds, I.S07.3472) were sown in 12 cm-diameter plastic pots filled with humus soil and vermiculite (about 7 to 1 ratio). For protoplast preparation, maize B73 were used. In a glasshouse, plants were kept in large cardboard boxes to avoid light, and the temperature was at ~ 17 °C (night) and ~ 28 °C (day) until the second leaves were fully expanded (~ 10–12 cm). To grow plants under normal sunlight, plants were cultivated under the same conditions, except that they were not enclosed in the cardboard boxes.

### Plasmid construction

The coding regions of the target genes were amplified from the cDNA synthesized from total RNA isolated from maize leaves (cv. B73) and cloned into the pM999 vector (kindly provided by Dr. Yongzhong Xing from the Huazhong Agricultural University). The primer sequences used for these cloning events are listed in the Additional file 1: Table S1. Plasmids were isolated using a Midi Extraction Kit (CWBiotech). The concentrations and quality of the plasmid DNA used for transfection were determined on a Nanodrop 2000 UV–Vis Spectrophotometer (ThermoFisher Scientific). The plasmid concentrations were adjusted to about 1 µg/µl and stored at − 20 °C until use.

For constructing plasmids expressing artificial miRNAs, a web-based tool (https://wmd3.weigelworld.org/cgibin/webapp.cgi) was adopted for the automated design of artificial microRNAs (amiRNAs). The cloning of amiRNA precursor fragment using the pRS300 vector followed Ossowski et al. [38]. The amiRNA precursor fragment include the pBSK multiple cloning site, so we subcloned the fragments of amiRNAs to the pM999 vector using the EcoRI and BamHI. Primers used to clone amiRNAs are listed in the Additional file 1: Table S2.

### Protoplast isolation and transformation

Protoplast isolation followed a protocol established for Arabidopsis [23] with modifications. The second well-expanded leaves were harvested from the base and sliced into leaf strips of 1–2 mm width transversely from the middle part of the leaves (the tips and bases were removed) using a fresh razor blade. The strips were immediately immersed into 15 ml of filter-sterilized enzyme solution [20 mM MES (morpholino-ethanesulfonic acid), pH 5.7, 0.6 M mannitol, 10 mM CaCl_2_, 0.1% BSA (bovine serum albumin; Sigma), 1.5% Cellulase R10 (Yakult Pharmaceutical), and 0.75% Macerozyme R10 (Yakult Pharmaceutical)] in a Petri dish. The samples were kept under vacuum (0.8 MPa) for 30 min in dark and then kept for 3–4 h at 25 °C in dark.

After digestion, 15 ml of W5 solution (4 mM MES, pH 5.7, 1.54 mM NaCl, 5 mM KCl, 125 mM CaCl_2_) was added to the enzyme solution containing the protoplasts. A cell strainer (70 µm; Corning) was wetted with 1–2 ml of W5 solution and the suspended protoplasts were filtered and transferred into a 50-ml sterilized centrifuge tube. The protoplasts were pelleted by centrifuging at 100*g* for 3 min at 4 °C, and then the pellet of protoplasts was resuspended with 5 ml of W5 solution and centrifuged again at 100*g* for 3 min at 4 °C. The pellet was resuspended in 1 ml of W5 buffer and kept on ice for 30 min.

Before initiating transfection, protoplasts were resuspended in the MMG buffer (4 mM MES, 0.4 M mannitol, 15 mM MgCl_2_) at 5 × 10^5^ cells/ml. For each transfection, 20 µl of plasmids (20 µg) was gently mixed with 100 µl of protoplasts in a 2-ml microcentrifuge tube, followed by adding an equal volume (120 μl) of PEG/Ca^2+^ solution [0.6 M mannitol, 100 mM CaCl_2_, and 40% PEG4000 (w/v) (Sigma)] and mixing by gentle inversion. The mixture was kept at 25 °C for 5–30 min before being diluted with 480 μl of W5 solution. After being centrifuged at 200*g* for 2 min, the supernatants were carefully removed by pipetting. Finally, the protoplasts were resuspended in 200 μl of WI (4 mM MES, pH 5.7, 0.6 M mannitol, 4 mM KCl) solution in a 2-ml microcentrifuge tube and incubated at 25 °C overnight in dark. After 12–16 h, protoplasts were collected by centrifugation at 200*g* for 2 min and the supernatants were removed by pipetting. The cells were immediately used for benzoxazinoid or RNA extraction or frozen in − 80 °C until extraction.

The plasmid pM999 carrying an *eGFP* (enhanced green fluorescence protein) gene was used as the positive control to determine transfection efficiency. Transfection efficiency was calculated as the percentage of fluorescent protoplasts to the total protoplasts, monitored under a fluorescent microscope (Leica DM5500 B).

### Extraction and analysis of benzoxazinoids

Transfected protoplasts were harvest at indicated times, and 200 µl of extraction solution [H_2_O:methanol:formic acid = 49.5:50:0.5, spiked with 10 µg of 4-methylumbelliferone (Sigma) per milliliter as the internal stand] was added to each protoplast sample. After 10 min of vortexing, samples were centrifuged at 15,000*g* for 10 min, and 90 µl of supernatants were transferred into the inserts in 2-ml HPLC glass vials. For extracting benzoxazinoids from maize leaves, 100 mg of ground leaf tissue was mixed with 1000 µl of extraction solution, and after vortexing for 10 min and subsequent centrifugation at 15,000*g* for 10 min, 400 μl of the supernatant was transferred to a 2-ml HPLC glass vial.

The analysis of benzoxazinoids was performed on an HPLC system equipped with a Shim-pack XR-ODS (2.0 mm I.D. × 75 mm L., 1.6 μm, Shim-pack) column coupled to a triple quadrupole mass spectrometer (LC-MS8040, Shimazu) with an electrospray ionization (ESI) source. Gradient elution was performed at 5–20% B over 3.0 min, 20–98% B over 3.0 min, holding at 98% B for 1 min, followed by re-equilibration at 5% A for 3.0 min, where A = 0.05% formic acid/water (v/v) and B = 0.05% formic acid/acetonitrile (v/v). The flow rate was 0.3 ml/min. The temperature of the column was maintained at 40 °C, and the injection volume was 5 µl. The ESI mass spectrometer was operated in a positive Q3 scan mode. The identities of DIMBOA-Glc, DIM_2_BOA-Glc, HDMBOA-Glc, HDM_2_BOA-Glc, and MBOA were confirmed with purified standards (kindly provided by Prof. Matthias Erb, University of Bern). The other benzoxazinoids were putatively identified by their molecular masses and comparing the chromatographic profiles of benzoxazinoids between the maize inbred line W22 (the wild-type background of *bx2::Ds* mutant) and the *bx2::Ds* mutant (GRMZM2G085661), which contains very little benzoxazinoids. The abundance of each metabolite was quantified from its peak area normalized by the area of internal stand. The acquired data were introduced to the SIMCA-P software package (v11.5, Umetric) for principal component analysis (PCA). The data were filtered by orthogonal signal correction (OSC) to remove variations from non-correlated factors. The detailed mass spectrometry conditions are shown in Additional file 1: Table S3.

### RNA extraction and quantitative real-time PCR analysis

Total RNA was extracted from maize protoplasts or leaves using the TRIzol reagent (Invitrogen) following the manufacturer’s instructions. The concentration of total RNA was measured on a NanoDrop 2000 spectrophotometer (ThermoFisher Scientific) and the integrity was examined on agarose gels. cDNA was synthesized from 500 ng of total RNA with RevertAid H Minus Reverse Transcriptase (ThermoFisher Scientific). Quantitative real-time PCR (qRT-PCR) was performed on a CFX Connect real-time system (Bio-Rad) using iTaqTM Universal SYBR Green Supermix kits (Bio-Rad). The maize *ZmGAPDH* (*glyceraldehyde-3-phosphate dehydrogenase*; GRMZM2G180625) served as the internal standard for normalizing the variation of cDNA concentrations. Four replicates from independently transfected protoplasts were used for qRT-PCR. Sequences of primers used for qRT-PCR are listed in Additional file 1: Table S4.

### Imaging of transfected protoplasts

Transfected protoplasts were observed under a research microscope equipped with fluorescence filters and light sources (Leica DM5500 B). For confocal imaging, protoplasts were imaged under a confocal laser scanning microscope (Olympus FV1000) for eGFP and chloroplast autofluorescence. Fluorescent signals were visualized using the following settings: eGFP, excitation wavelength = 488 nm, emission wavelengths = 520–560 nm; chlorophyll autofluorescence: excitation wavelength = 633 nm, emission wavelength = 680–700 nm.

### E-box motif analysis

The 2-kb sequences upstream of translation initiation sites of benzoxazinoid biosynthetic genes were retrieved from the Phytozome (https://phytozome.jgi.doe.gov/pz/portal.html). The E-box motif sequence (5ʹ-CANNTG-3ʹ) was used to search against these sequences using the online Global Align tool at the NCBI website.

## Results

### Transfection of maize protoplasts and analysis of benzoxazinoids

**Fig. 2:**
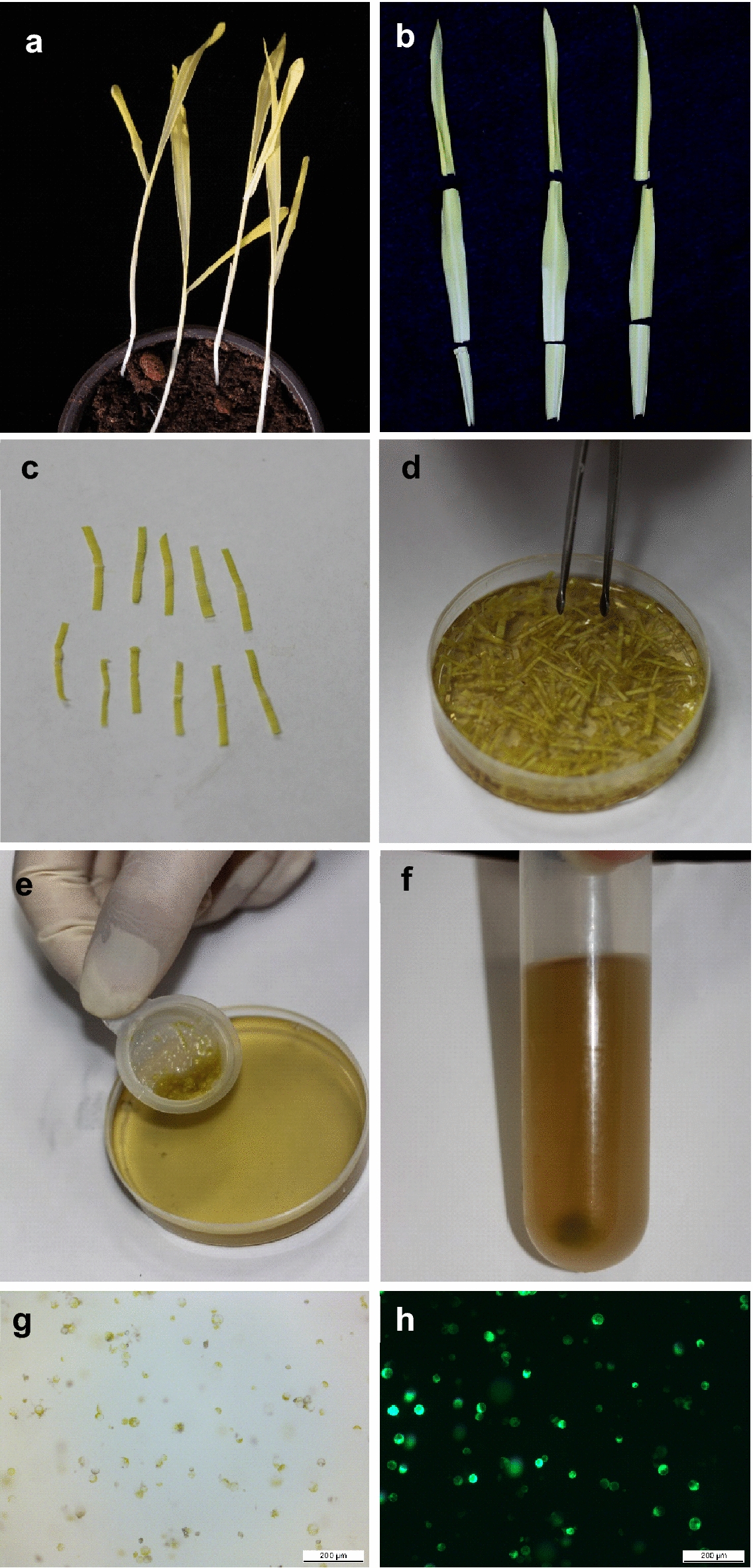
Isolation and transfection of protoplasts from etiolated maize leaves. **a** Etiolated 12-day-old maize leaves. **b** The middle leaf segments of etiolated maize seedlings. **c** Leaf strips. **d** Leaf strips in protoplast isolation solution. **e** Removal of leaf strip debris by filtering through a 70-μm cell strainer. **f** Washing and collecting the maize protoplasts. **g**, **h** Bright-field (**g**) and fluorescence images (**h**) of the same maize protoplasts expressing eGFP 12 h after transfection. Scale bars = 200 μm

Preparation of high-quality maize protoplasts is essential for successful transformation and subsequent analyses. We used leaves of healthy etiolated maize, which were grown in soil for about 12–15 days (Fig. 2a). The middle regions of the second fully expanded leaves (Fig. 2b) were sliced into approximately 1-mm strips and digested with the enzyme solution containing cellulose RS and macerozyme R10 (Fig. 2c, d). The released protoplasts were filtered through a 70-μm mesh (Fig. 2e), and then transferred into a 50-ml sterilized centrifuge tube to wash and collect protoplasts (Fig. 2f). A typical preparation (10 leaves) could yield 1 to 5 × 10^6^ cells. Most of the protoplasts are spherical, indicating that they remain intact (Additional file 1: Fig. S1). A previous study has shown that the optimal ratio between the amount of DNA and number of protoplasts was 100 µg of DNA to 2 to 3 × 10^5^ protoplasts for each transfection [39]. Thus, 20 µg of DNA was used for 5 × 10^4^ cells for each transfection following a method modified from Yoo, Cho and Sheen [23]. The plasmid pM999 carrying *eGFP* (pM999-eGFP) was used to examine the efficiency of transfection. It was found that in this manner, usually more than 50% of protoplasts showed strong eGFP fluorescence 12 h after transfection (Fig. 2g, h).

Standards of benzoxazinoid DIMBOA-Glc, DIM_2_BOA-Glc, HDMBOA-Glc, HDM_2_BOA-Glc and MBOA, which were purified from maize, were individually injected to the HPLC–MS system to optimize the HPLC (a 10 min gradient program) and MS conditions. For DHBOA, DIBOA-Glc, DIBOA, DIMBOA, DIM_2_BOA, M_2_BOA, HMBOA-Glc and HM_2_BOA-Glc, whose standards were not available, the inferred mass-to-charge ratios (molecular masses + 1, since the ionized forms are the protonated molecules) were used to scan the total extract obtained from maize leaves, so as to putatively identify these benzoxazinoids, and the MS running conditions were respectively optimized using these putatively identified benzoxazinoids (chromatogram and MS profiles see Additional file 1: Fig. S2). Next, the extract from *bx2::Ds* (GRMZM2G085661) mutant in the W22 genetic background, which has very little benzoxazinoids, was run on the HPLC–MS under the optimized condition. Compared with the extract from W22 maize, all the chromatographic peak areas of benzoxazinoids greatly decreased in the sample of *bx2::Ds* extract (Additional file 1: Fig. S3), well supporting the identities of these benzoxazinoids. Next, we tested whether the sensitivity of HPLC–MS allows well detection of benzoxazinoids in protoplasts. pM999-eGFP was transfected to 5 × 10^4^ maize protoplasts, and after 16 h, the protoplasts were extracted with 200 µl of extraction solution. The extract was run on HPLC–MS. Under our HPLC–MS condition, all benzoxazinoids showed large peak areas, even after tenfold dilution of the protoplast extraction, the peaks of benzoxazinoids still exhibited large signal/noise ratios (data not shown), indicating that the sensitivity of the HPLC–MS system well met the requirement of benzoxazinoid quantification in protoplasts. Thus, a 10-min-run HPLC–MS method was established for detecting maize benzoxazinoids (see “Materials and methods” and Additional file 1: Table S3 for details).

### Overexpressing and silencing the benzoxazinoid biosynthetic gene *ZmBX1* in maize protoplasts

**Fig. 3:**
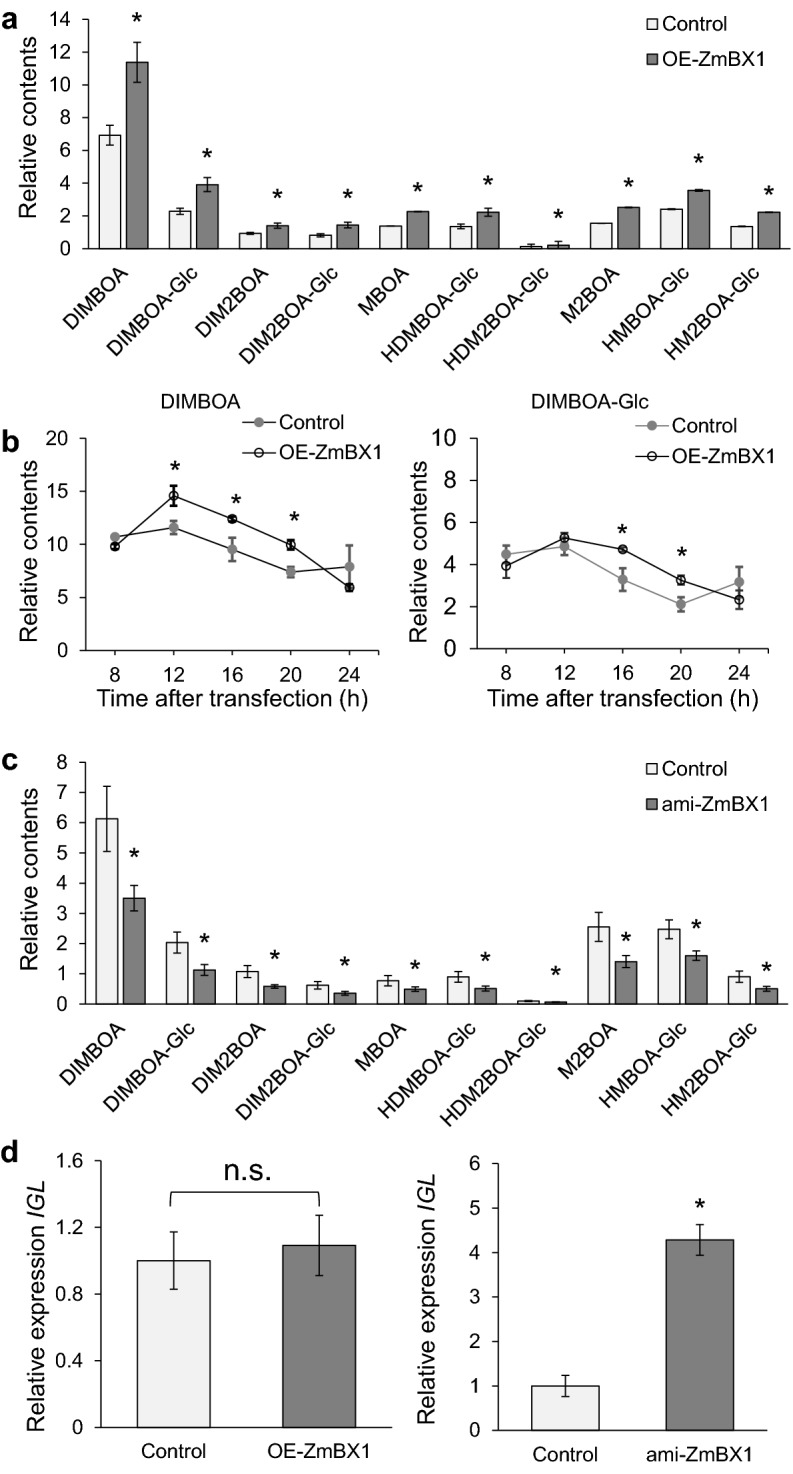
Changes of benzoxazinoid contents induced by overexpressing and silencing *ZmBX1* in maize protoplasts. Maize protoplasts were transfected with pM999-eGFP as the control, and simultaneously, maize protoplasts were transfected with pM999-ZmBX1 or pM999-ami-ZmBX1 to overexpress or silence *ZmBX1* (OE-ZmBX1 and ami-ZmBX1, respectively). **a** Relative benzoxazinoid abundances in control and OE-ZmBX1 maize protoplasts. **b** The accumulation patterns of two main benzoxazinoids, DIMBOA and DIMBOA-Glc in control and OE-ZmBX1 maize protoplasts at different times after transfection. **c** Relative contents of benzoxazinoids in control and ami-ZmBX1 protoplasts. **d** The relative expression levels of *ZmIGL* transcript in OE-ZmBX1 and ami-ZmBX1 protoplasts. Data are means ± SE. Asterisks indicate significant differences between control and overexpression or gene-silenced protoplasts (Student’s t-test; n = 5; *P ≤ 0.05)

The maize *ZmBX1* gene encodes a key enzyme in the initial steps of benzoxazinoid biosynthesis, catalyzing the formation of free indole from indole-3-glycerol phosphate [14]. *bx1* mutant exhibits decreased benzoxazinoid content [8] and overexpression of *ZmBX1* enhances the levels of benzoxazinoids [17]. To determine whether transient manipulation of *ZmBX1* expression levels in maize protoplasts is sufficient to change the contents of benzoxazinoids, we first overexpressed *ZmBX1* by transfecting maize protoplasts with pM999-ZmBX1, which carries the *ZmBX1* coding region driven by a CaMV35S promoter. Twelve h after transfection, compared with the empty vector, transfection of pM999-ZmBX1 led to 20-fold increase in the *ZmBX1* expression level (Additional file 1: Fig. S4) and ~ 50% increased concentration of DIMBOA (Fig. 3a). This result was similar to what was found in the transgenic maize plants overexpressing *ZmBX1* gene, in which the average content of DIMBOA was up to 1.8 mM in the transgenics, compared with 0.7 mM in the wild-type maize plants [17].

Given that the accumulation of secondary metabolites usually lags behind the increase of transcripts of biosynthetic enzyme genes, we transfected maize protoplasts with the plasmid pM999-ZmBX1 and the protoplasts were harvested at 8, 12, 16, 20, and 24 h to determine the optimum duration of benzoxazinoid accumulation after transfection. Although different benzoxazinoids showed somewhat different kinetics of accumulation, most of them had the highest concentrations at 12 or 16 h (Fig. 3b and Additional file 1: Fig. S5). Thus, 16 h of incubation time post transfection was adopted for all further experiments.

Artificial microRNA (amiRNA) has become a powerful tool to knock down the transcript levels of target genes, and transfecting Arabidopsis and rice protoplasts with amiRNAs successfully achieved gene silencing with high efficiencies [40]. The short lengths (21 to 24 nt) of amiRNAs also minimize the chance of off-target silencing and make amiRNA constructs easy to prepare. To examine whether amiRNA-mediated gene silencing can be used in maize protoplasts to study benzoxazinoid-related biosynthetic genes or regulators, we first created an amiRNA-expressing construct specifically targeting *ZmBX1*, which was named pM999-ami-ZmBX1, and transfected it to maize protoplasts. pM999-ami-ZmBX1 was able to efficiently decrease 80% of the *ZmBX1* transcripts (Additional file 1: Fig. S6). Consistently, most of the benzoxazinoids showed about 50% decreased concentrations (Fig. 3c). *ZmIGL* (*indole-3-glycerol phosphate lyase*) gene is a homolog of *ZmBX1*, both of which are evolutionarily related to the *tryptophan synthase alpha subunit* [41]. We found that the transcript level of *ZmIGL* was more than fourfold greater in maize protoplasts transfected with pM999-amiRNA-ZmBX1 than in those transfected with pM999-eGFP, whereas overexpression of *ZmBX1* in maize protoplasts did not affect the *ZmIGL* transcript abundance (Fig. 3d). Thus, these amiRNA constructs specifically targeted *ZmBX1* but not the homolog gene *ZmIGL*, and it is possible that there is a balancing regulation on the transcription of *ZmBX1* and *ZmIGL*, enabling transcript compensation between these two genes.

Thus, combining maize mesophyll protoplast transfection and HPLC–MS, this system can be used for rapid analysis of the functions of benzoxazinoid biosynthetic genes.

### Functional analyses of transcription factors in regulation of benzoxazinoid biosynthesis in maize protoplasts

Thus far, little is known about how benzoxazinoid biosynthesis is regulated. Recently, RNA-seq analyses and metabolomic comparisons indicated a large number of transcription factors (TFs) are differentially regulated in the maize Mo17 and B73, which are two inbreed lines with high and low benzoxazinoid contents, respectively [12]. Co-expression analysis suggested that four TFs, *ZmMYB61* (GRMZM2G108959), *ZmNAC35* (GRMZM2G179049), *ZmGRAS* (GRMZM2G015080), and *ZmWRKY* (GRMZM2G425430) might play roles in benzoxazinoid regulation [20]. Moreover, herbivore feeding on a maize leaf also increased the benzoxazinoid concentrations in distal/systemic leaves, and RNA-seq analysis indicated that multiple TFs might be involved in the spatial regulation of benzoxazinoid concentrations in maize plants [42]. Thus, we selected three TFs, the *ZmMYB61* (GRMZM2G108959) [20], *ZmbHLH20* (GRMZM2G414252) and *ZmbHLH76* (GRMZM2G112629) [42], from these two transcriptome studies, and took advantage of this maize protoplast transfection system to examine whether this system could be used in identifying regulators of benzoxazinoid biosynthesis.

**Fig. 4:**
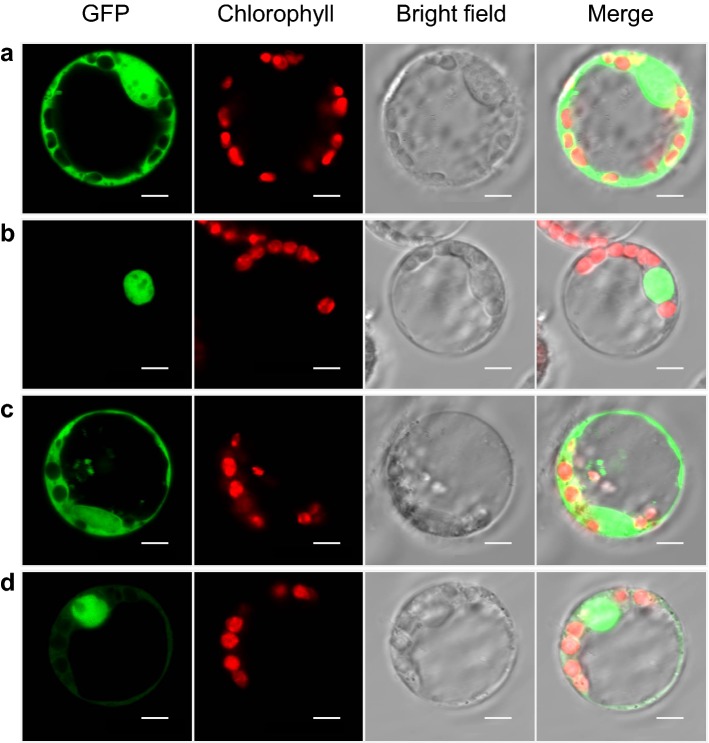
Subcellular localization analysis of ZmbHLH20 and ZmbHLH76 in maize protoplasts. Maize protoplasts were transfected with *eGFP* (**a**), OsGhd7 nucleus-targeting sequence fused with *eGFP* (**b**), *ZmbHLH20-eGFP* (**c**), or *ZmbHLH76-eGFP* (**d**). Left to right: eGFP fluorescence channel, chloroplast fluorescence channel, bright-field channel, and merge. Bars = 5 μm

First, an experiment was performed to determine the subcellular localization of the fusion proteins ZmbHLH20-eGFP and ZmbHLH76-eGFP. In order to examine whether our system is suitable for determination of protein subcellular locations, we individually transfected protoplasts with pM999-eGFP and pM999-OsGhd7-eGFP, the latter of which carries the *eGFP* gene fused after a nucleus-targeting sequence [43]. eGFP fluorescence was distributed throughout the nuclei and cytoplasm of protoplasts transfected with pM999-eGFP (Fig. 4a), and the eGFP fluorescence from cells transfected with pM999-OsGhd7-eGFP was seen in the nuclei (Fig. 4b). For ZmbHLH20-eGFP, fluorescence was restricted to the cell nuclei and cytoplasm (Fig. 4c). Transient expression of pM999-ZmbHLH76-eGFP in maize protoplasts indicated that ZmbHLH76 mainly localized in the nuclei but a small portion was in the cytoplasm (Fig. 4d).

**Fig. 5:**
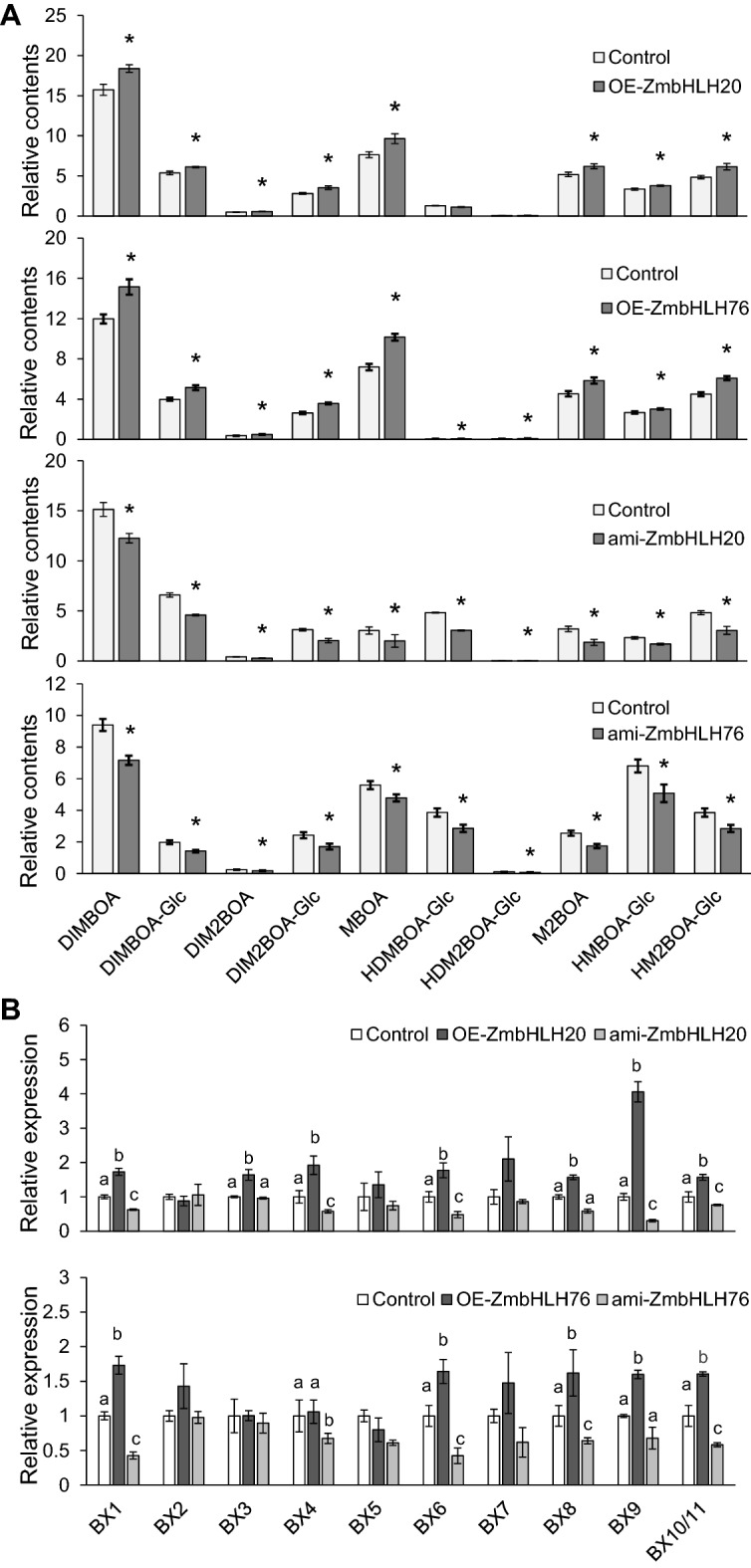
Analysis of the functions of ZmbHLH20 and ZmbHLH76 in regulating benzoxazinoids in maize protoplasts. **A** The change of benzoxazinoid contents induced by overexpressing and silencing *ZmbHLH20* and *ZmbHLH76* in maize protoplasts. **B** Transcript levels of *BX1*, *BX2*, *BX3*, *BX4*, *BX5*, *BX6*, *BX7*, *BX8*, *BX9*, and *BX10*/*11* in maize protoplasts after overexpression or silencing of *ZmbHLH20* and *ZmbHLH76*. Protoplasts transformed with empty vector were used as controls. Data are means ± SE. The letters a, b and c indicate significant differences among control, overexpression and gene-silenced protoplasts (ANOVA analysis; n = 4; *P ≤ 0.05)

Next, these TF genes were overexpressed in maize protoplasts and the resulting changes of benzoxazinoid concentrations were quantified on the HPLC–MS system. While transfecting maize protoplasts with pM999-ZmMYB61 overexpressed *ZmMYB61* transcripts 60 times (Additional file 1: Fig. S7), the contents of benzoxazinoids in maize protoplasts were not altered (Additional file 1: Fig. S8). Thus, *ZmMYB61* was excluded from further analysis. In contrast, overexpression of *ZmbHLH20* and *ZmbHLH76* increased their expression levels 8- and 20-fold, respectively (Additional file 1: Fig. S9), and the contents of protoplast benzoxazinoids were also elevated: DIMBOA, DIMBOA-Glc, DIM_2_BOA-Glc, MBOA, M_2_BOA, HM_2_BOA-Glc exhibited about 20% increase after overexpression of *ZmbHLH20,* and DIMBOA, DIMBOA-Glc, DIM_2_BOA-Glc, MBOA, HDMBOA-Glc M_2_BOA, HMBOA-Glc, HM_2_BOA-Glc exhibited about 20% increase after overexpression of *ZmbHLH76* (Fig. 5A). To further confirm the function of *ZmbHLH20* and *ZmbHLH76* in regulating benzoxazinoid biosynthesis, gene silencing of *ZmbHLH20* and *ZmbHLH76* was achieved by transfecting maize protoplasts with plasmids expressing the gene-targeting amiRNAs. The target genes *ZmbHLH*20 and *ZmbHLH*76 were both about 60% silenced (Additional file 1: Fig. S9). In agreement with the data of benzoxazinoid contents in protoplasts overexpressing *ZmbHLH20* and *ZmbHLH76*, we found that most of the benzoxazinoids showed approximately 20% decreased concentrations (Fig. 5A). qRT-PCR analysis indicated that most of the benzoxazinoid biosynthesis genes were up-regulated in the protoplasts overexpressing *ZmbHLH20* and *ZmbHLH76* and down-regulated in the protoplasts silenced in *ZmbHLH20* and *ZmbHLH76* (Fig. 5B), suggesting that *ZmbHLH20* and *ZmbHLH76* directly or indirectly regulates the transcript levels of these benzoxazinoid biosynthesis genes. bHLH TFs carry a DNA-binding region that binds to a consensus core element known as the E-box (5ʹ-CANNTG-3ʹ) [44]. Indeed, sequence analysis indicated the existence of possible E-box binding sites in the promoter sequences of benzoxazinoid biosynthesis genes, *BX1, BX2, BX3, BX4, BX5, BX6, BX7, BX8, BX9, BX10,* and *BX11* (Additional file 1: Fig. S10).

These data indicate that *ZmbHLH20* and *ZmbHLH76* likely function as positive regulators of multiple benzoxazinoid biosynthesis genes and thus influence the accumulation of benzoxazinoids. Importantly, these data demonstrate that this maize protoplast transfection and HPLC–MS system is suitable for rapid analysis of gene functions, including TFs.

## Discussion

In maize, benzoxazinoids are a class of important defensive metabolites against insects and pathogens [8], whose biosynthesis pathway has largely been identified. However, very little is known about the regulatory network of benzoxazinoid biosynthesis [10, 45]. Although forward genetic and transcriptomic studies have provided a number of candidate genes possibly involved in benzoxazinoid biosynthesis or regulation [17, 20, 42, 46, 47], the functions of these candidate genes still require in vitro and/or in vivo studies. Here, we show that the maize protoplast transfection-HPLC–MS system can be exploited as a rapid and relatively high-throughput platform for screening genes function in benzoxazinoid biosynthesis or regulation, before these genes are studied in planta. Given the lengthy and low-efficient maize transformation, we expect that this rapid screening system will greatly facilitate the research in identification of new benzoxazinoid biosynthesis and catabolism genes and regulators. Gene cloning can be done within 1 week, plasmid preparation and protoplast transfection can be finished in 3 days, and HPLC–MS analysis can be done within a day (10 min run/sample). If large-scale high-through cloning techniques, such as the Gateway cloning, are used and etiolated maize seedlings are grown regularly, tens of genes can be studied within a week.

*ZmBX1* gene has long been identified to catalyze the initial step of benzoxazinoid biosynthesis [14]. Indeed, transfecting maize protoplasts with constructs to overexpress or silence *ZmBX1* resulted in increased and decreased levels of most benzoxazinoids, respectively (Fig. 3a, c). Moreover, based on transcriptomic data, we examined three TFs, which are putative regulators of benzoxazinoids. Our system showed that *ZmMYB61* very likely does not control benzoxazinoid biosynthesis (Additional file 1: Fig. S8), while overexpressing and silencing *ZmbHLH20* and *ZmbHLH76* resulted in elevated and reduced benzoxazinoid contents, respectively (Fig. 5A). The expression levels of some of the benzoxazinoid biosynthesis genes were indeed up-regulated after *ZmbHLH20* and *ZmbHLH76* over-expression and down-regulated after *ZmbHLH20* and *ZmbHLH76* were silenced (Fig. 5B). These data suggest that these two TFs are important candidate regulators of benzoxazinoid biosynthesis.

Furthermore, maize protoplast system can be easily used for determination of protein subcellular localizations, as demonstrated by the experiment for localizations of ZmbHLH20 and ZmbHLH76 (Fig. 4), in which we found that ZmbHLH20-eGFP was distributed in both nuclei and cytoplasm and ZmbHLH76-eGFP was mainly localized in the nuclei but a small portion of ZmbHLH76-eGFP was in the cytoplasm. Although many TFs are exclusively distributed in nuclei, some can be found outside of nuclei, such as cytoplasm and cell membranes. Upon perception of certain development- or stress-related cues, these cell membrane- or cytoplasm-located TFs can be posttranslationally modified (e.g., phosphorylated) and thereafter translocated into nucleus to activate the transcription of their target genes. Some TFs need to bind to other proteins, including TFs, before they can enter the nucleus. For example, in *Arabidopsis* phosphorylation of a NAC transcription factor NTL6 by SnRK2.8 in the cytoplasm is required for the nuclear import of NTL6 [48].

## Conclusions

Here we present a high-throughput method which combines maize protoplast transfection and HPLC–MS analysis, allowing rapid screening for the regulatory and biosynthetic genes of maize benzoxazinoids. The same maize protoplast transfection protocol can also be used to study protein subcellular localization, protein–protein interactions, and protein–DNA interactions. If plants and constructs are available, all these experiments can be accomplished within a few days.

We also propose that this protoplast transfection-HPLC/GC–MS system can be modified to suit for studying other types of metabolites, even volatile compounds, given that appropriate extraction method (e.g., solid phase microextraction, SPME) and gas chromatography (GC)–MS, instead of HPLC–MS, is used for the quantification. Importantly, this approach can also be used for other plant species, including many crops or wild plants whose transformation is difficult or not available. Even for model plants, such as rice and Arabidopsis, which are amenable to transformation and have sufficient mutant resources, protoplast transfection-HPLC or GC–MS (or MS/MS, which usually has greater sensitivity than does MS) system could be utilized as a practical system for studying or screening the candidate genes in metabolite biosynthesis and regulation, before genetically modified plants are prepared or used.

## Supplementary information

**Additional file 1: Table S1.** Primers used in gene cloning and vector construction. **Table S2.** Primers used for amiRNA plasmid construction. **Table S3.** HPLC–MS running parameters. **Table S4.** Primers used for RT-qPCR analysis of gene expression. **Fig. S1.** Image of isolated maize protoplasts. **Fig. S2.** The HPLC chromatogram and the MS profiles of all the benzoxazinoids. **Fig. S3.** Benzoxazinoid levels in W22 and bx2::Ds transposon knockout mutants. **Fig. S4.** *ZmBX1* transcript level in maize protoplasts after overexpressing target gene. **Fig. S5.** The contents of HDMBOA-Glc, MBOA, M2BOA, and DIM2BOA-Glc in protoplasts transfected with ZmBX1 over time. **Fig. S6.** *ZmBX1* transcript level in maize protoplasts after silencing with gene-specific amiRNA. **Fig. S7.** *ZmMYB61* transcript level in maize protoplasts after overexpressing target gene. **Fig. S8.** The changes of benzoxazinoid contents induced by overexpressing ZmMYB61. **Fig. S9.** Transcription levels of target genes after overexpression or silencing. **Fig. S10.** Schematic representation of the predicted bHLH protein binding motifs (E-boxes) in the promoters of benzoxazinoid biosynthesis genes.

## Data Availability

All data generated in this study are included in this article and additional files. Material is available from the corresponding author on reasonable request.
