# Supplementary material for: An efficient system composed of maize protoplast transfection and HPLC–MS for studying the biosynthesis and regulation of maize benzoxazinoids
Source: Plant Methods. 2019 Nov 28;15:144. doi: 10.1186/s13007-019-0529-2 (PMC6882228; doi:10.1186/s13007-019-0529-2)
Supplement: Supplementary file 1 — Additional file 1: Table S1. Primers used in gene cloning and vector construction. Table S2. Primers used for amiRNA plasmid construction. Table S3. HPLC–MS running parameters. Table S4. Primers used for RT-qPCR analysis of gene expression. Fig. S1. Image of isolated maize protoplasts. Fig. S2. The HPLC chromatogram and the MS profiles of all the benzoxazinoids. Fig. S3. Benzoxazinoid levels in W22 and bx2::Ds transposon knockout mutants. Fig. S4. ZmBX1 transcript level in maize protoplasts after overexpressing target gene. Fig. S5. The contents of HDMBOA-Glc, MBOA, M2BOA, and DIM2BOA-Glc in protoplasts transfected with ZmBX1 over time. Fig. S6. ZmBX1 transcript level in maize protoplasts after silencing with gene-specific amiRNA. Fig. S7. ZmMYB61 transcript level in maize protoplasts after overexpressing target gene. Fig. S8. The changes of benzoxazinoid contents induced by overexpressing ZmMYB61. Fig. S9. Transcription levels of target genes after overexpression or silencing. Fig. S10. Schematic representation of the predicted bHLH protein binding motifs (E-boxes) in the promoters of benzoxazinoid biosynthesis genes. [file 13007_2019_529_MOESM1_ESM.pdf]

Table S1 Primers used in gene cloning and vector construction

**Gene**	**Forward Primer (5′ - 3′)**	**Reverse Primer (5′ - 3′)**
ZmBX1	CTAGCTCGAGCATGGCTTTCGCGCCCAAAAC (XhoI)	GACTGGATCCCATATGCGTACTGTAGTTTTTATTCAATC (BamHI)
ZmMYB61	TCTAGAGTATGGGGAGGCCACCGTG (XbaI)	TCTAGAGACGATACAGCAACATGAAC (XbaI)
ZmbHLH20	GAATTCGCACCGTAACCTGATGGAGTTC (EcoRI)	GGATCCTCGTGTTCGTCACAGCATGTCG (BamHI)
ZmbHLH20-eGFP	GAATTCGCACCGTAACCTGATGGAGTTC (EcoRI)	TCTAGATCGTGTTCGCAGCATGTCGG (XbaI)
ZmbHLH76	GAATTCGTCGATCGAGATGAAGAGCCG (EcoRI)	GGATCCCTCAGCTCAGACGAGGATCG (BamHI)
ZmbHLH76-eGFP	GAATTCGTCGATCGAGATGAAGAGCCG (EcoRI)	TCTAGACTCAGCGACGAGGATCGTC (XbaI)

Note: The underlined sequences are the restriction enzyme digestion sites, and the corresponding enzymes are indicated in the brackets.

Table S2 Primers used for amiRNA plasmid construction

**Primer name**	**Prime sequence (5′ - 3′)**
amiRNA-ZmBX1-I	gaTATCGAATGAACGGCCCGCTAtctctcttttgtattcc
amiRNA-ZmBX1-II	gaTAGCGGGCCGTTCATTCGATAtcaaagagaatcaatga
amiRNA-ZmBX1-III	gaTAACGGGCCGTTCTTTCGATTtcacaggtcgtgatatg
amiRNA-ZmBX1-IV	gaAATCGAAAGAACGGCCCGTTAtctacatatatattcct
amiRNA-ZmbHLH20-I	gaTCTATGGTAACGATCGGTCTAtctctcttttgtattcc
amiRNA-ZmbHLH20-II	gaTAGACCGATCGTTACCATAGAtcaaagagaatcaatga
amiRNA-ZmbHLH20-III	gaTAAACCGATCGTTTCCATAGTtcacaggtcgtgatatg
amiRNA-ZmbHLH20-IV	gaACTATGGAAACGATCGGTTTAtctacatatatattcct
amiRNA-ZmbHLH76-I	gaTATACTAGAGTACTACTCCGCtctctcttttgtattcc
amiRNA-ZmbHLH76-II	gaGCGGAGTAGTACTCTAGTATAtcaaagagaatcaatga
amiRNA-ZmbHLH76-III	gaGCAGAGTAGTACTGTAGTATTtcacaggtcgtgatatg
amiRNA-ZmbHLH76-IV	gaAATACTACAGTACTACTCTGCtctacatatatattcct
pRS300-A	CTGCAAGGCGATTAAGTTGGGTAAC
pRS300-B	GCGGATAACAATTTCACACAGGAA ACAG

Note: pRS300-A and pRS300-B are primers used for amplifying the amiRNA precursors and thereafter cloning into the pM999 vector by digestion with EcoRI and BamHI. The lowercase sequences match with the plasmid pRS300 sequences and the uppercase sequences are used to replace the sequences in the pRS300 vector with the target amiRNA sequences by overlapping PCR.

Table S3 HPLC-MS running parameters

**Benzoxazinoid**	**Retention time (min)**	**$(M+H)^+/Z$**	**Mode**
DHBOA-Glc	3.521	365.95	Q3 SIM(+)
DIBOA	3.563	182.05	Q3 SIM(+)
DHBOA	4.648	182.05	Q3 SIM(+)
DIBOA-Glc	4.672	344	Q3 SIM(+)
M_2BOA	4.868	196	Q3 SIM(+)
HMBOA-Glc	4.942	380.1	Q3 SIM(+)
DIMBOA-Glc	5.107	373.95	Q3 SIM(+)
DIMBOA	5.208	212	Q3 SIM(+)
DIM_2BOA	5.236	241.95	Q3 SIM(+)
MBOA	5.766	166	Q3 SIM(+)
HDM_2BOA-Glc	5.834	440	Q3 SIM(+)
HDMBOA-Glc	5.851	388	Q3 SIM(+)
DIM_2BOA-Glc	5.863	405	Q3 SIM(+)

Table S4 Primers used for RT-qPCR analysis of gene expression

**Gene**	**Accession Number**	**Primer (5′ - 3′)**
ZmIGL	GRMZM2G046191	Forward: GCCTCATAGTTCCCGACCTC<br>Reverse: GAATCCTCGTGAAGCTCGTG
ZmBX1	GRMZM2G085381	Forward: CCTGCTCGGACCCCTACAT<br>Reverse: GGACCCCCGCCTCTTTCAT
ZmBX2	GRMZM2G085661	Forward: GACGAGGACGACGATAAGGACTT<br>Reverse: GGCCATACTCCTTCTGAAGAGACAG
ZmBX3	GRMZM2G167549	Forward: TCCACATGAAGGGCAAGGAC<br>Reverse: ATCTCCATGGTCGCGAATCC
ZmBX4	GRMZM2G172491	Forward: TGCTCGCGAACCTCATCTAC<br>Reverse: GAAGCGTCATCCCGAACTGA
ZmBX5	GRMZM2G063756	Forward: AGATCATGCTCGCCAACCTC<br>Reverse: GAACGTCTCGTCCATGCTCA
ZmBX6	GRMZM6G617209	Forward: TTCTTCAACACGGACGTGAG<br>Reverse: GCCATCGAGTCCTATGGTGT
ZmBX7	GRMZM2G441753	Forward: CCAGCCACGACCCCGCCAAGG<br>Reverse: AGTAGTGTTCTCCTTCGAGGCGCCG
ZmBX8	GRMZM2G085054	Forward: CAGCTGGAGAGAGGGGAGAT<br>Reverse: TCCTCTTCCTGATGCCCTCC
ZmBX9	GRMZM2G161335	Forward: GCCAGCTGCGACGCCCCCTTCA<br>Reverse: CGGTACGCCATGTAGTCGC
ZmBX10/11	GRMZM2G311036<br>GRMZM2G336824	Forward: CTGCAACCGCTGTTTTCCTC<br>Reverse: CCGTGGAGATATGGCTTGCT
ZmGAPDH	GRMZM2G180625	Forward: AGCAGGTCGAGCATCTTCG<br>Reverse: CTGTAGCCCCACTCGTTGTC
ZmbHLH20	GRMZM2G414252	Forward: CGATATCCCCGATGCAGACC<br>Reverse: CTTCTTTGGGTACCCGGTGG
ZmbHLH76	GRMZM2G112629	Forward: TTCAAGCTGCACGAGGTCAT<br>Reverse: GCGAGTGGACGGTGTAGAAT
ZmMYB61	GRMZM2G108959	Forward: GTGGACCAACTACCTGAGGC<br>Reverse: GGAGGTAGGAGGCTATGGCT

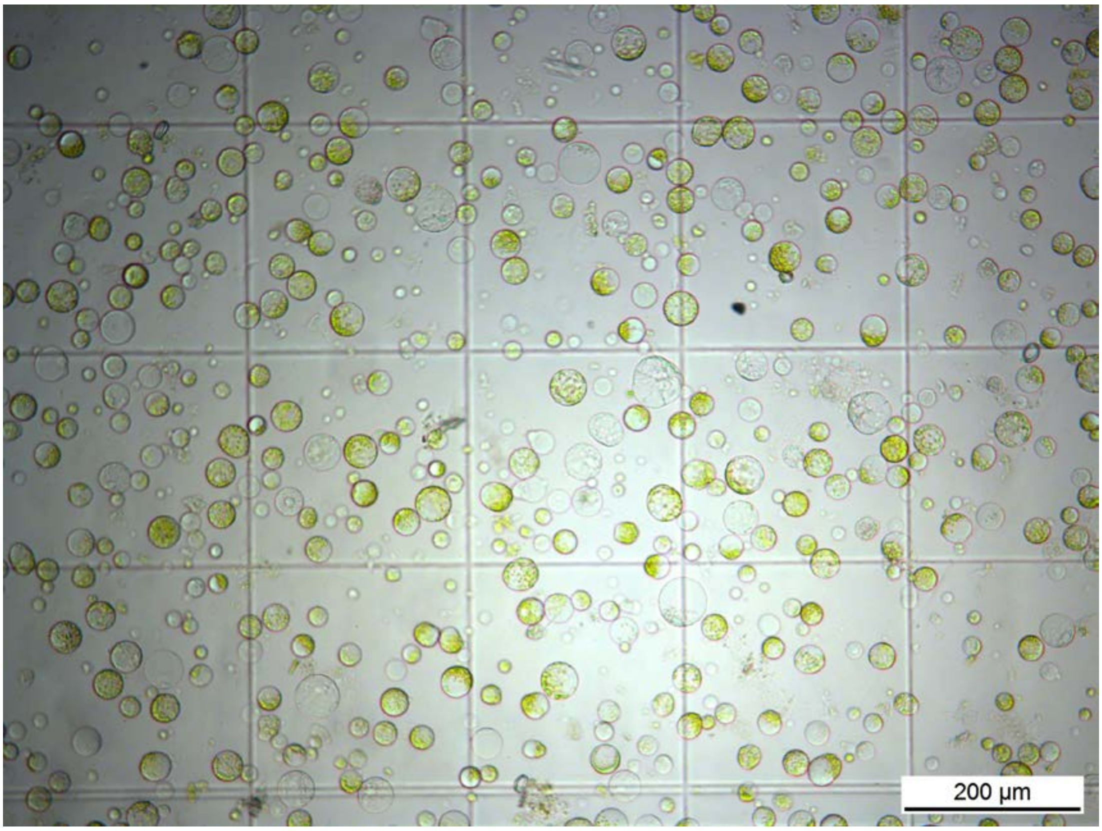

Fig. S1. Image of isolated maize protoplasts.

Isolated maize protoplasts were imaged with a microscope under a 100 × magnification. Scale bar = 200 µm.

A

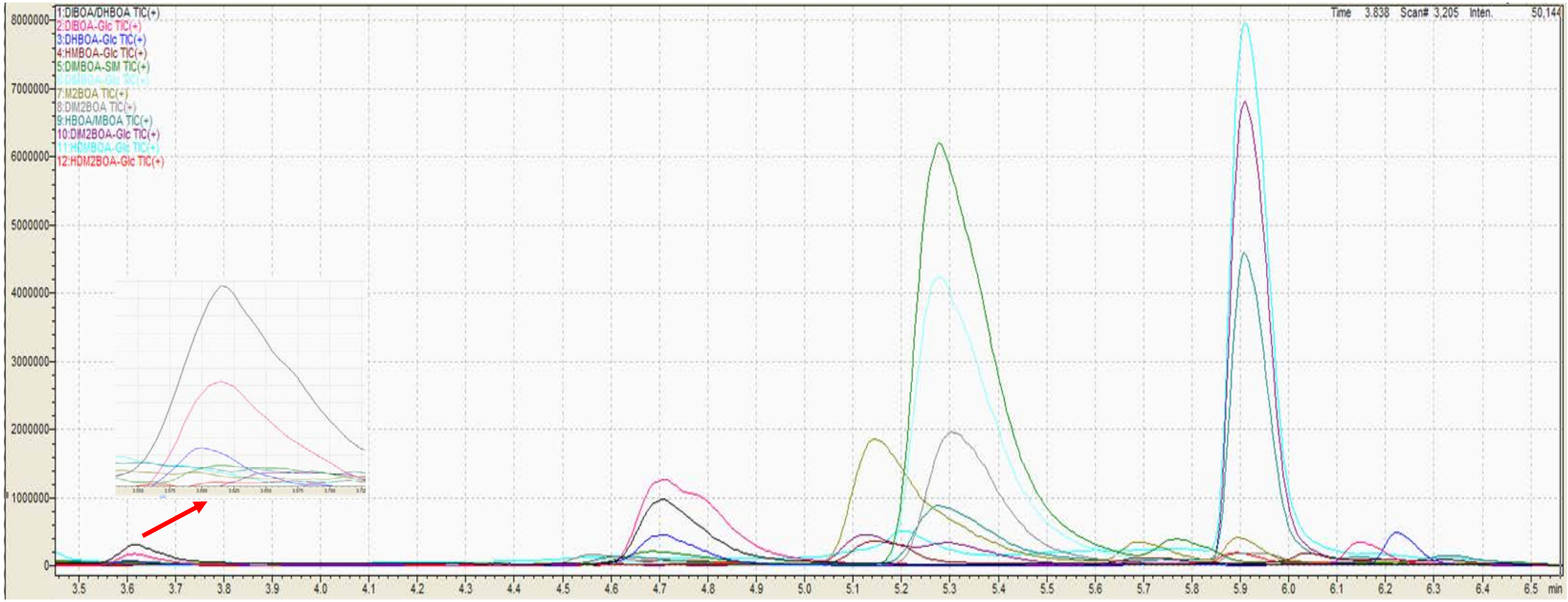
1:DIBOA/DHBOA TIC(+)
2:DIBOA-Glc TIC(+)
3:DHBOA-Glc TIC(+)
4:HMBOA-Glc TIC(+)
5:DIMBOA-SIM TIC(+)
7:M2BOA TIC(+)
8:DIM2BOA TIC(+)
9:HBOA/MBOA TIC(+)
10:DIM2BOA-Glc TIC(+)
11:HDMBOA-Glc TIC(+)
12:HDM2BOA-Glc TIC(+)
Time 3.838 Scan# 3,205 Inten.
8000000
7000000
6000000
5000000
4000000
3000000
2000000
1000000
0
3.5 3.6 3.7 3.8 3.9 4.0 4.1 4.2 4.3 4.4 4.5 4.6 4.7 4.8 4.9 5.0 5.1 5.2 5.3 5.4 5.5 5.6 5.7 5.8 5.9 6.0 6.1 6.2 6.3 6.4 6.5 min

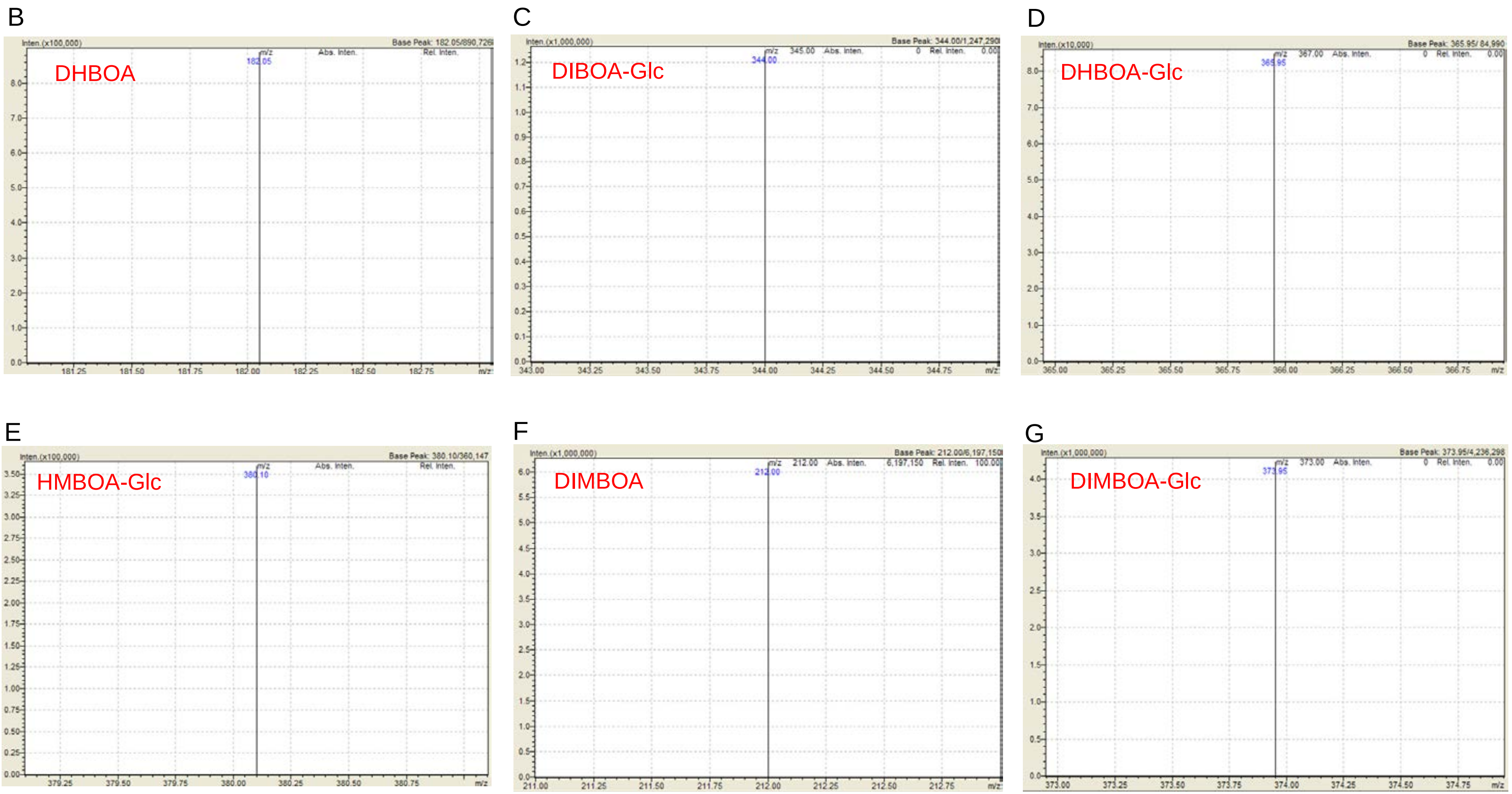

B
DHBOA
Base Peak: 182.05/890,726
182.05
C
DIBOA-Glc
Base Peak: 344.00/1,247,290
344.00
D
DHBOA-Glc
Base Peak: 365.95/ 84,990
365.95
E
HMBOA-Glc
Base Peak: 380.10/360,147
380.10
F
DIMBOA
Base Peak: 212.00/6,197,150
212.00
G
DIMBOA-Glc
Base Peak: 373.95/4,236,298
373.95

H

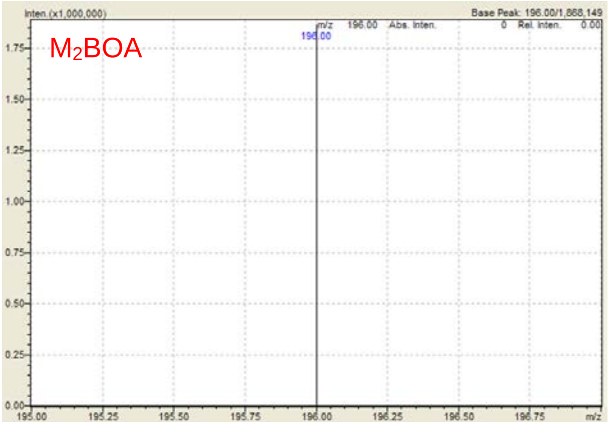
M2BOA
Inten.(x1,000,000)
Base Peak: 196.00/1,868,149
196.00

I

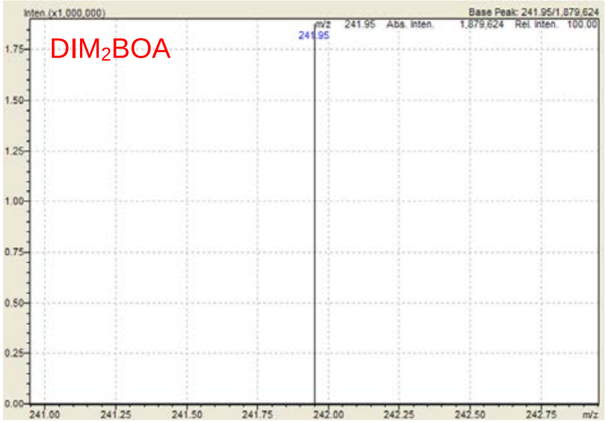
DIM2BOA
Inten.(x1,000,000)
Base Peak: 241.95/1,879,624
241.95

J

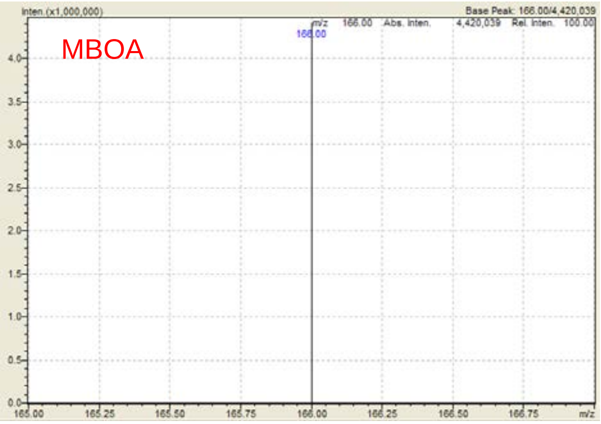
MBOA
Inten.(x1,000,000)
Base Peak: 166.00/4,420,039
166.00

K

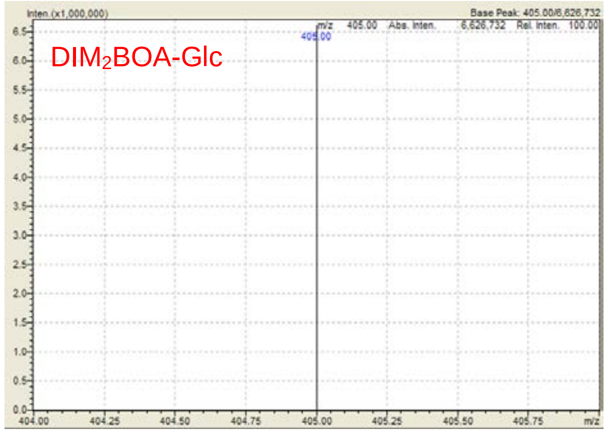
DIM2BOA-Glc
Inten.(x1,000,000)
Base Peak: 405.00/6,626,732
405.00

L

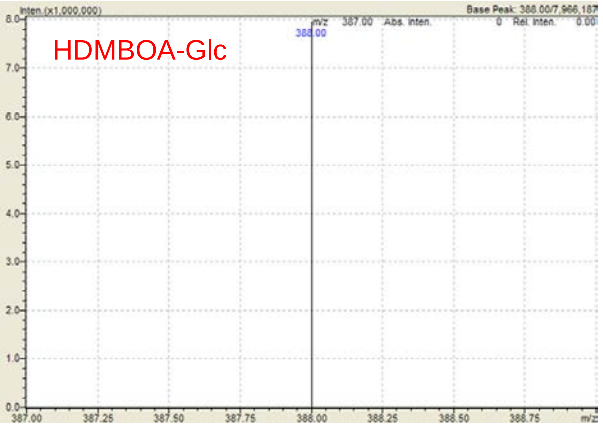
HDMBOA-Glc
Inten.(x1,000,000)
Base Peak: 388.00/7,966,187
388.00

M

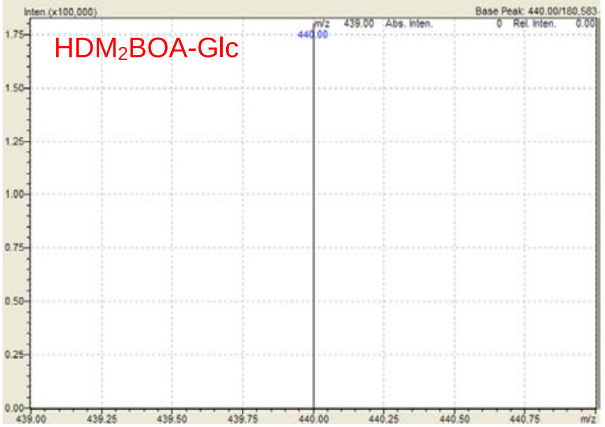
HDM2BOA-Glc
Inten.(x100,000)
Base Peak: 440.00/180,583
440.00

N

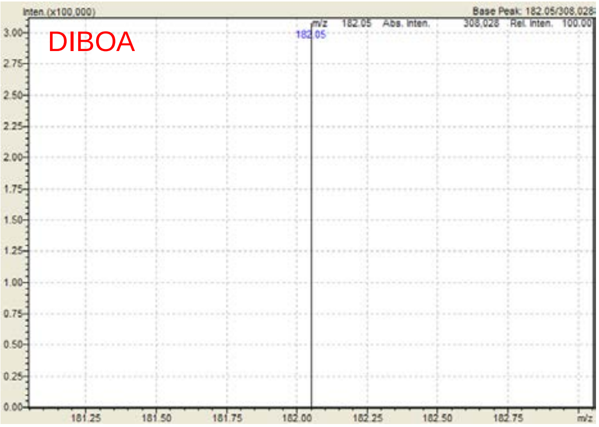

Fig. S2. The HPLC chromatogram and the MS profiles of all the benzoxazinoids.

(A) The HPLC chromatogram of the benzoxazinoids; (B-N) The MS profiles of the benzoxazinoids, (B) DHBOA, (C) DIBOA-Glc, (D) DHBOA-Glc, (E) HMBOA-Glc, (F) DIMBOA, (G) DIMBOA-Glc, (H) M_2BOA, (I) DIM_2BOA, (J) MBOA, (K) DIM_2BOA-Glc, (L) HDMBOA-Glc, (M) HDM_2BOA-Glc, (N) DIBOA. Y-axis indicates ionic intensity; X-axis indicates peak time.

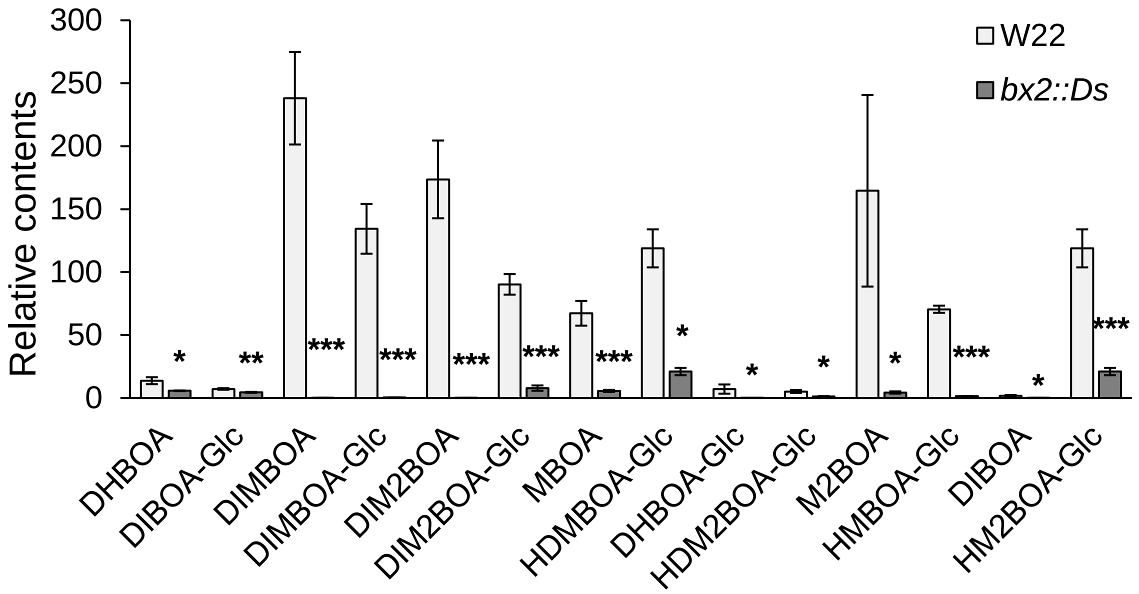

Fig. S3. Benzoxazinoid levels in W22 and *bx2::Ds* transposon knockout mutants.

Maize W22 and *bx2::Ds* mutants were grown until two-leaves stage. Leaves were harvested and benzoxazinoids were extracted for HPLC-MS analysis. Data are means ± SE. Asterisks indicate significant differences between WT and *bx2::Ds* (Student's t-test; n = 4; *, P ⩽ 0.05, **, P ⩽ 0.01, ***, P ⩽ 0.001).

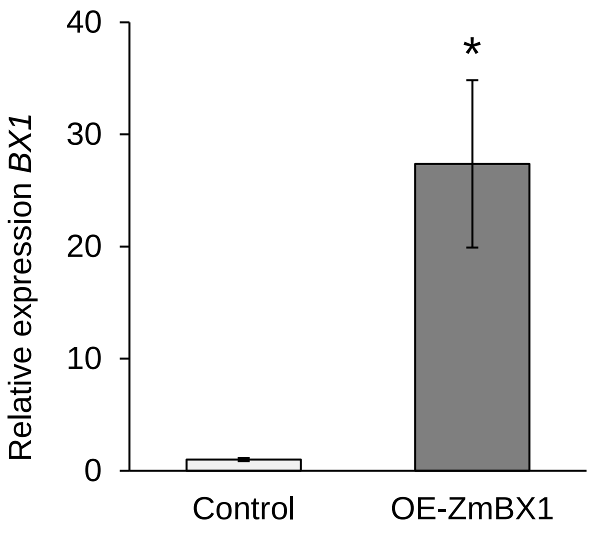

Fig. S4. *ZmBX1* transcript level in maize protoplasts after overexpressing target gene. Transcript values in controls are normalized to 1. Data = means ± SE. Asterisks indicate significant differences between control and overexpression or gene-silenced protoplasts (Student's t-test; n = 4; *, P ⩽ 0.05).

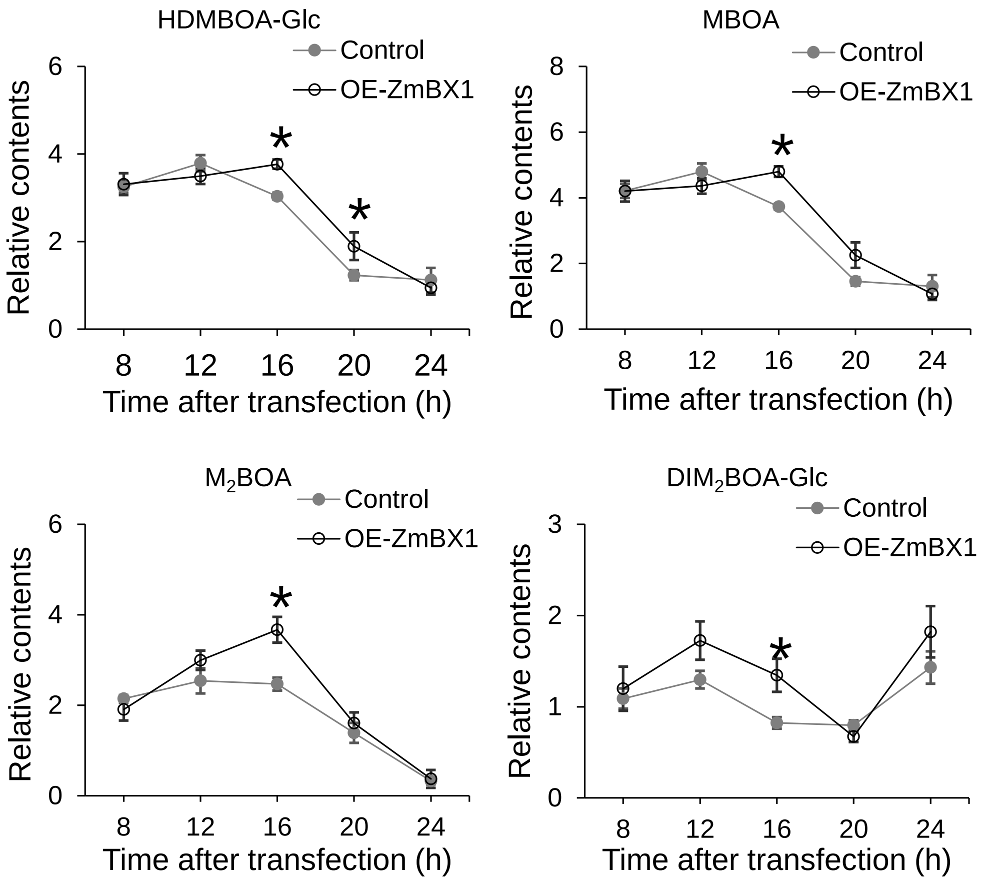

Fig. S5. The contents of HDMBOA-Glc, MBOA, M$_2$BOA, and DIM$_2$BOA-Glc in protoplasts transfected with *ZmBX1* over time.

Maize protoplasts were transfected with pM999-eGFP as the control or with pM999-ZmBX1 to overexpress or silence ZmBX1 (OE-ZmBX1). The relative contents of four main benzoxazinoids, HDMBOA-Glc, MBOA, M2BOA, and DIM2BOA-Glc in control and OE-ZmBX1 maize protoplasts at different times were quantified. Asterisks indicate significant differences between control and OE-ZmBX1 protoplasts (Student's t-test; n = 5; *, $P \leqslant 0.05$).

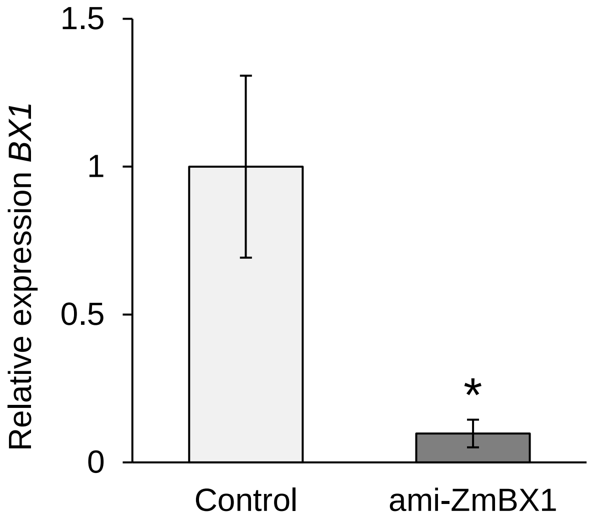

Fig. S6. *ZmBX1* transcript level in maize protoplasts after silencing with gene-specific amiRNA.

Transcript values in controls are normalized to 1. Data = means ± SE. Asterisks indicate significant differences between control and overexpression or gene-silenced protoplasts (Student’s t-test; n = 4; *, P ⩽ 0.05).

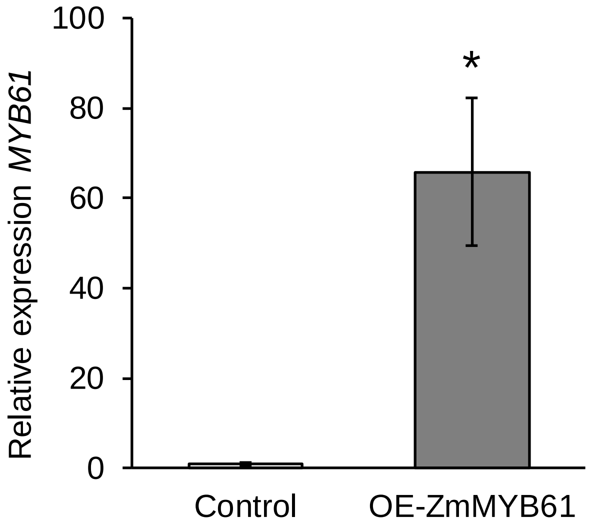

Fig. S7. *ZmMYB61* transcript level in maize protoplasts after overexpressing target gene.

Transcript values in controls are normalized to 1. Data = means ± SE. Asterisks indicate significant differences between control and overexpression or gene-silenced protoplasts (Student's t-test; n = 4; *, P ⩽ 0.05).

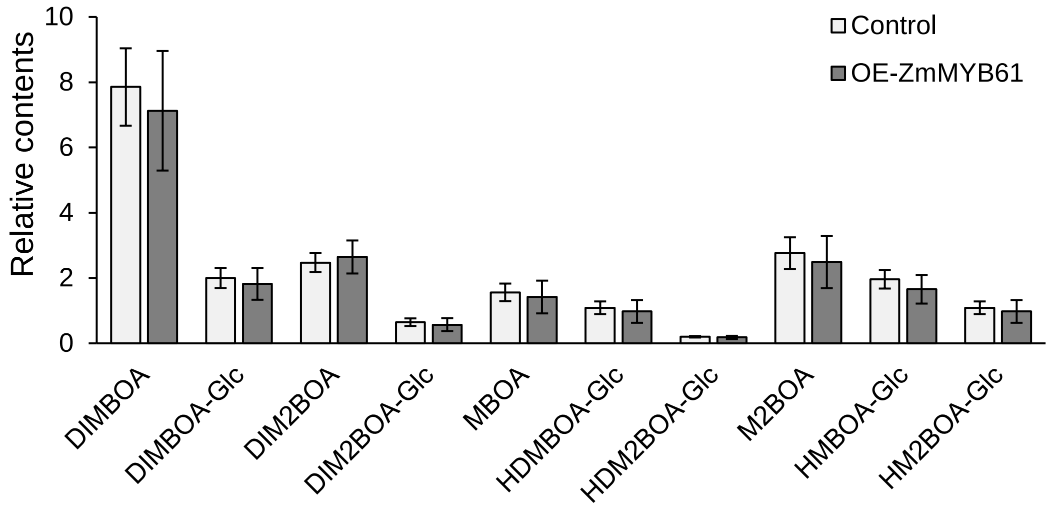

Fig. S8. The changes of benzoxazinoid contents induced by overexpressing *ZmMYB61*. Maize protoplasts were transfected with pM999-ZmMYB61 (OE-ZmMYB61) or pM999-eGFP as the control. There are no significant differences between control and ZmMYB61-overexpression protoplasts (Student’s t-test; n = 5).

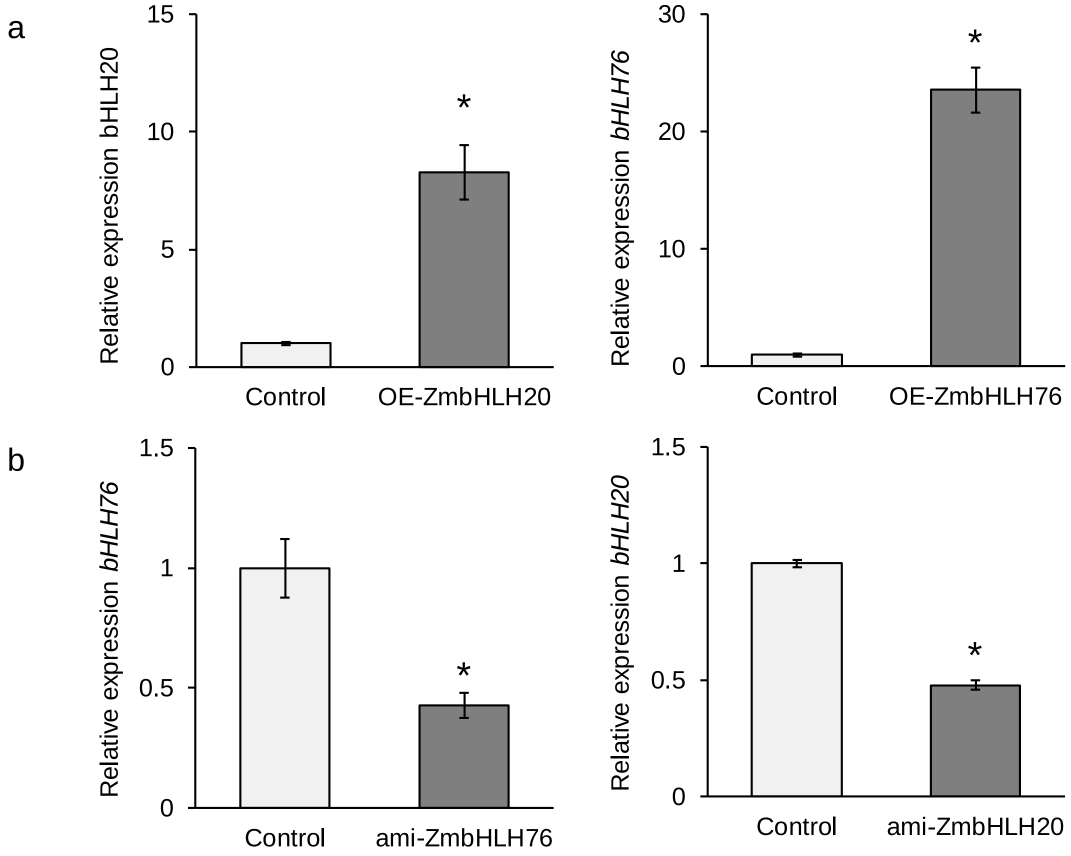

Fig. S9. Transcription levels of target genes after overexpression or silencing.

Maize protoplasts were transfected with empty vector (control) or vectors for overexpression of *ZmbHLH20*, and *ZmbHLH76* (OE-ZmbHLH20, and OE-ZmbHLH76, respectively) or silencing *ZmbHLH20*, and *ZmbHLH76* (ami-ZmbHLH20 and amiZmbHLH76, respectively). (a) *ZmbHLH20*, and *ZmbHLH76* transcript levels in maize protoplasts after overexpression. (b) *ZmbHLH76* and *ZmbHLH20* transcript levels in maize protoplasts after silencing with gene-specific amiRNAs. Transcript values in controls are normalized to 1. Data = means ± SE. Asterisks indicate significant differences between control and overexpression or gene-silenced protoplasts (Student’s t-test; n = 4; *, P ⩽ 0.05).

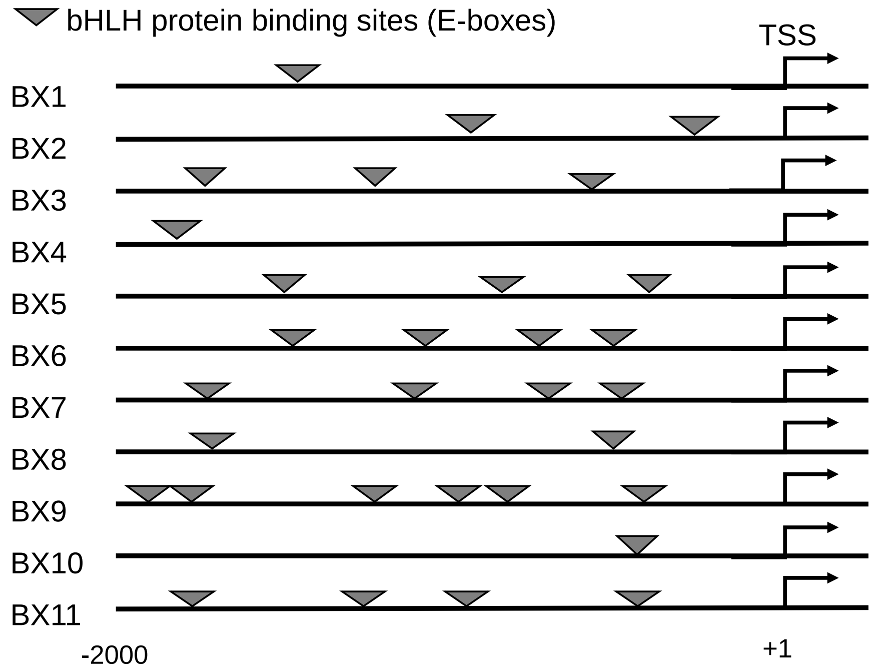

Fig. S10. Schematic representation of the predicted bHLH protein binding motifs (E-boxes) in the promoters of benzoxazinoid biosynthesis genes.

Two-kb regions upstream of the translation start sites (TSS, +1) was used for predicting the E-boxes, which are indicated by the arrow heads.
